# Acceleration of the Deamination of Cytosine through Photo-Crosslinking

**DOI:** 10.3390/cimb45060298

**Published:** 2023-05-29

**Authors:** Siddhant Sethi, Yasuharu Takashima, Shigetaka Nakamura, Licheng Wan, Nozomi Honda, Kenzo Fujimoto

**Affiliations:** Bioscience, Biotechnology, and Biomedical Engineering Research Area, Japan Advanced Institute of Science and Technology, Asahi-dai 1-1, Ishikawa, Nomi 923-1292, Japan; siddhant@jaist.ac.jp (S.S.);

**Keywords:** DNA manipulation, cytosine deamination, photo-crosslinking, nucleobase editing, RNA editing

## Abstract

Herein, we report the major factor for deamination reaction rate acceleration, i.e., hydrophilicity, by using various 5-substituted target cytosines and by carrying out deamination at high temperatures. Through substitution of the groups at the 5′-position of the cytosine, the effect of hydrophilicity was understood. It was then used to compare the various modifications of the photo-cross-linkable moiety as well as the effect of the counter base of the cytosine to edit both DNA and RNA. Furthermore, we were able to achieve cytosine deamination at 37 °C with a half-life in the order of a few hours.

## 1. Introduction

Genetic disorders are caused by mutations in the genome. Many of these genetic disorders are due to a single base mutation in the otherwise perfect sequence [1,2,3]. Leigh syndrome is such an example wherein a single point mutation of base 8993 from thymine to cytosine in the mitochondrial DNA leads to a life-threatening condition due to loss of muscle control [4,5]. Available treatment for such diseases includes certain drugs and diet therapy which are able to treat the disease symptomatically but not completely [6]. To treat these genetic disorders, it is important to focus on the root cause and edit the genome artificially [7,8].

Although there are various enzymatic methods for targeting DNA and manipulate it, the practical application of these methods is limited [9,10]. Considering these drawbacks, many advanced techniques to carryout genome editing have been developed and are being used extensively, such as zinc finger nuclease, transcription activator-like effector-based nuclease, clustered regularly interspaced short palindromic repeats-associated system (CRISPR-Cas system), and many others [11,12,13,14,15]. CRISPR-Cas system has become the most advanced enzymatic method for genetic manipulation [16]. However, there are possibilities of off-target effects [17]. In addition, there are reports of other enzymatic methods such as RNA-specific adenosine [18] and cytosine deaminases [19], along with chemical methods such as bisulfite conversion [20]; however, they also have some limitations such as non-specific deamination and non-applicability for in vivo applications.

The limitations of the chemical methods of nucleobase editing led to the development of photochemical methods. Some of these methods use psoralen and azobenzene incorporated in the artificial DNA strand and could undergo photo-crosslinking or photo-ligation for the anti-sense effects [21,22,23,24,25]. Back in 2010, our group reported a novel compound, 3-cyanovinylcarbazole (^CNV^K). This compound was able to crosslink with pyrimidine specifically using UV radiation of 366 nm and was also able to undergo photo-splitting, reversing the crosslinking reaction using 312 nm irradiation [26]. Furthermore, we also reported that ^CNV^K can be incorporated into oligo-DNA (ODN) and can then be crosslinked to cytosine in the complementary strand to achieve enzyme-free highly specific pin-point nucleic acid editing through photo-crosslinking with the target cytosine and leading to the deamination of the target cytosine [26,27].

While incorporated in the ODN, ^CNV^K can form a cross-link with cytosine in the complementary strand at the −1 position via a [2 + 2] cycloaddition reaction in a sequence-specific manner [27]. This leads to the loss of aromaticity of the cross-linked cytosine, thus making it labile for nucleophilic attack by water leading to the deamination of the cytosine [28]. The major limitation of this method was the use of high temperatures to afford the deamination reaction.

For the in vivo applications of photo-crosslink-induced cytosine deamination, we carried out the reaction under physiological conditions. We have previously shown that hydrogen bonding contributes to the rate of deamination of the cross-linked cytosine, but the overall conversion yield was low, and the half-life of the deamination reaction was in the order of multiple days at 37 °C [29]. We further correlated the effect of the hydrophilicity of the cross-linker with the rate of deamination [30,31], as it has been shown in multiple reports that the rate of cytosine deamination can be accelerated in vivo with regard to the neighboring bases of the cytosine pyrimidine dimer (CPD) [32,33,34].

In this report, we have discussed how the hydrophilicity of the target cytosine can affect the rate of deamination reactions at high temperatures due to the varying hydrophilicity of the target cytosine by changing the substituents at the 5-position. Furthermore, upon establishing the role of hydrophilicity at high temperatures, we went on to report the effect of the hydrophilic environment around the target cytosine by tweaking the counter base and photo-crosslinker in both DNA and RNA. Furthermore, we then studied the effect of highly increased hydrophilicity around the target cytosine by dis-joining the photo-active ODNs and modifying the counter-base with a free phosphate group to achieve deamination at 37 °C within a few hours.

## 2. Results and Discussion

**Figure 1:**
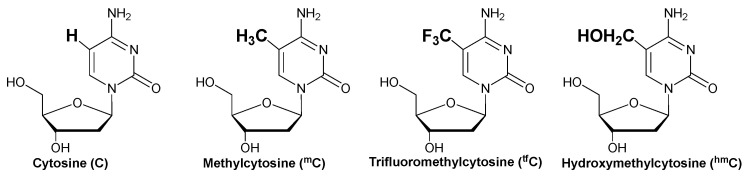
Structure of the cytosine derivatives.

The derivatives of cytosine, 5-methyl cytosine (^m^C), and 5-hydroxymethyl cytosine (^hm^C) have been extensively studied for their role in epigenetic regulation [35,36]. Furthermore, fluorine substitution has substantial effects on the biological properties of molecules, including lipophilicity [37]. Thus, in this study, the target cytosines of 3-cyanovinylcarbazole (^CNV^K) were modified to ^m^C, ^hm^C, and trifluoromethyl cytosine (^tf^C) (Figure 1) to understand the effects of these modifications on the ^CNV^K cross-linked deamination reaction’s efficiency.

**Figure 2:**
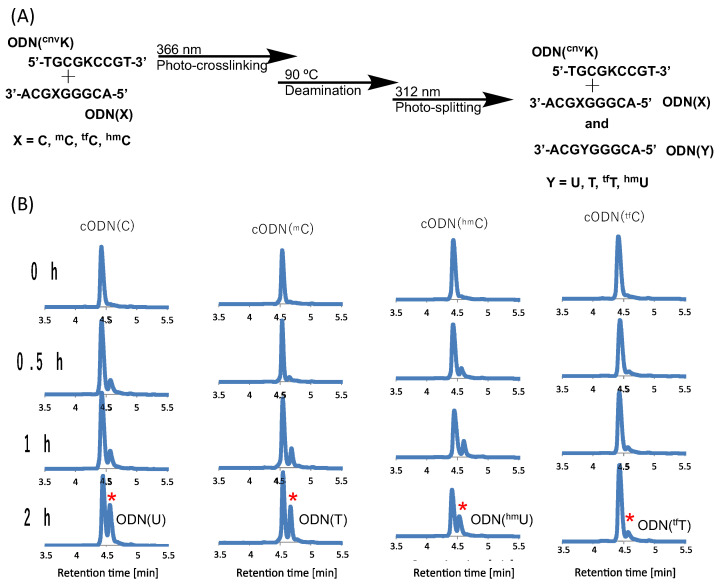
(**A**) Scheme of the photo-crosslink-assisted cytosine deamination reaction using cytosine derivatives. (**B**) UPLC analysis of the deamination reaction of the photo-cross-linked duplex consisting of ODN(K) and cODN(C) consisting of cytosine derivatives. [ODN(K<>C)] = 5 μM in 50 mM Na-cacodylate buffer (pH 7.4) containing 100 mM NaCl. incubated at 37 °C; photo splitting was performed with a transilluminator (312 nm) at 37 °C. The peak marked with an asterisk (*) indicates the newly formed product as depicted on each plot.

The oligo-DNA (ODN) incorporated with ^CNV^K and modified cytosines was synthesized using an automated DNA synthesizer (NTS M-2-MIX_NPS) with the phosphoramidites of ^CNV^K and ^tf^C were synthesized as per the literature [37], while the other phosphoramidites were purchased from Glen research. After purification using reverse-phase HPLC, the ODNs were photo-crosslinked using 385 nm UV radiation. Furthermore, for the deamination reaction, the cross-linked products were incubated at 90 °C and then photo-split using 312 nm radiation for 15 min (Figure 2A).

Upon deamination and then photo-splitting, the respective deaminated products were analyzed using ultra-high performance liquid chromatography (UPLC) with a gradient set to separate the original target ODN with cytosine (and its derivatives) and the newly formed uracil and other derivatives, respectively. The UPLC chromatograms show the formation of a new peak with a slight delay to that of the target ODN post incubation but with differing intensity (Figure 2B).

Before high-temperature incubation, the UPLC chromatograms showed only a single peak for each of the target ODNs but as the incubation time increases, a new peak starts emerging for each of the modified cytosines. It is interesting to note here that in the case of C, the new peak marked with a red asterisk shows the highest intensity after 2 h incubation, while in the case of ^tf^C, the new peak is lowest in intensity compared to the other modifications.

**Figure 3:**
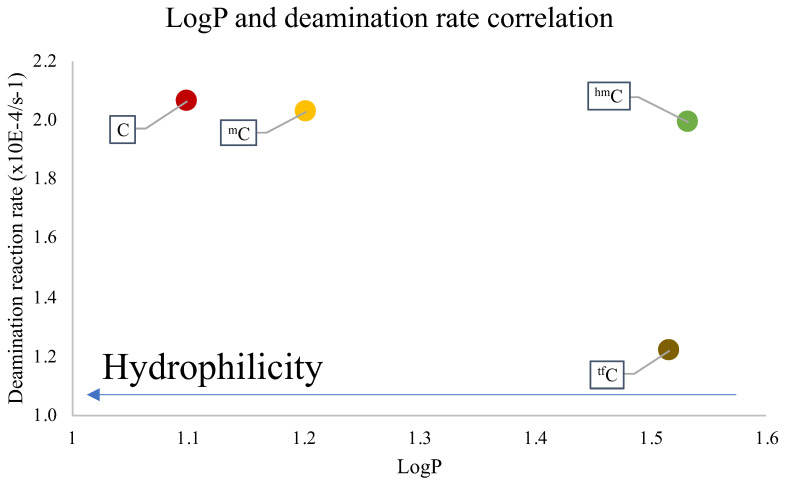
Dependence of LogP and the reaction rate constant of the deamination reaction at 90 °C.

To understand the disparity in the rate of the deamination reaction, the hydrophilicity of each of the cross-linked ODNs was measured using the water-octanol partition coefficient (LogP) measurement method [38,39], wherein the water-phase sample was analyzed with reverse-phased HPLC analysis (Figure S1). It was observed that the cross-linked ODN with cytosine has the highest hydrophilicity, while the ODN with ^tf^C is mostly lipophilic. Upon comparing the results of LogP and the deamination rate, it was ascertained that the most hydrophilic ODN, i.e., the one containing the unmodified cytosine, has the highest rate of deamination and vice-versa (Figure 3).

These results are in agreement with our previous reports that the hydrophilic micro-environment of the target cytosine is a major factor which affects the rate of deamination of not only the cytosine but also the derivates of the cytosine. This further confirms that the effect of hydrophilicity is not only important for deamination under physiological conditions but at higher temperatures as well, therefore confirming the importance of using a hydrophilic micro-environment around the deamination target.

In order to further understand the role of hydrophilicity in the deamination of the photo-crosslinked cytosine under physiological conditions, we studied the rate of the deamination reaction by tweaking the micro-environment of the target cytosine by changing the counter base from guanine to inosine and cytosine and studied them in combination with the crosslinkers ^CNV^K, 3-hydroxyvinylcarbazole (^OHV^K), 3-amidevinylcarbazole (^NH2V^K), and 3-methoxyvinylcarbazole (^OMeV^K) in the oligo-RNA (ORN). Previously, we have reported that by changing the counter base and photo-crosslinker in the ODN, a higher deamination rate can be achieved using a highly hydrophilic combination [31].

**Figure 4:**
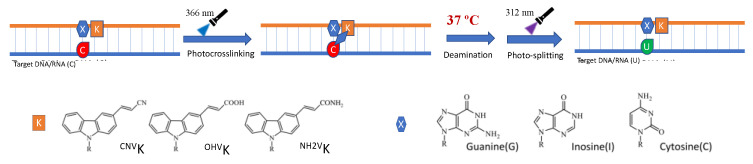
Scheme of deamination of cytosine with different counter base and photo-crosslinker incorporated in the ODN.

We followed the same protocol, wherein the ORN containing the target cytosine was crosslinked to the ODN containing the photo-crosslinker and the counter base using 385 nm UV radiation followed by incubation at 37 °C for 7 days and then photo-splitting of the crosslinked ODN/ORN duplex using 312 nm UV light (Figure 4).

The crosslinked ODN(XK)<>ORN(C) (5 µM) were incubated for 7 days at 37 °C in cacodylate buffer (50 mM) with 100 mM sodium chloride (pH 7.4) and then photo-split using 312 nm radiation for 15 min at 37 °C. The photo-split ORNs were then analyzed by UPLC. The deamination of the cytosine which afforded uracil as its product was studied by comparing the peak areas in the UPLC chromatogram of both the original peak (ORN(C)) and new peak (ORN(U)) after incubation (Figures S2–S4). With inosine as the counter base and by varying the crosslinker, it was found that the highest rate of deamination was with ODN(I^OHV^K) followed by ODN(I^CNV^K), ODN(I^NH2V^K), and ODN(^OMeV^K). Similar trends were observed in the case of using counter bases G and C as well. While comparing the rate of deamination among different counter bases, it was found that inosine was able to accelerate the deamination rate to the highest extent within the three counter bases followed by guanine and cytosine with the corresponding crosslinker.

This led us to the conclusion that among the counter bases, inosine has the highest ability to accelerate the deamination of the target cytosine in the RNA, and among the photo-crosslinkers, the maximum efficiency of deamination was observed with ^OHV^K.

**Figure 5:**
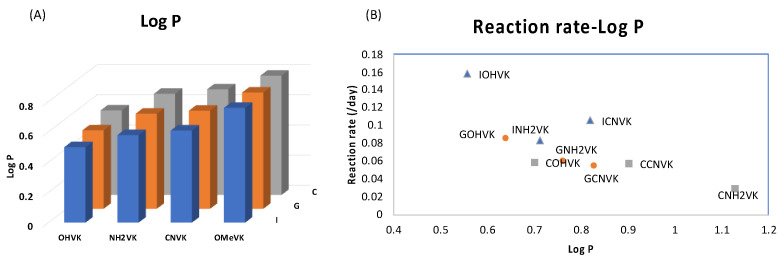
(**A**) Bar graph representing the LogP of each combination of ODN containing a counter base (I, G, or C) and a photo-crosslinker (^OHV^K, ^OMeV^K, ^CNV^K, or ^NH2V^K). The detailed data are provided in Table S5. Lower LogP values represent higher hydrophilicity. (**B**) Correlation of LogP and the reaction rate constant of the deamination reaction at 37 °C.

**Figure 6:**
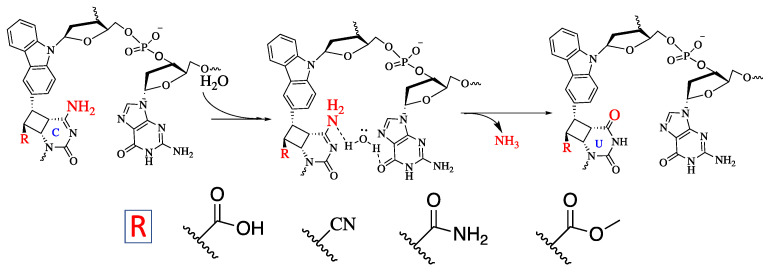
Proposed mechanism of the deamination of cytosine cross-linked to the cross-linker in the complementary strand.

Further, we then tried to find the relationship between the rate of deamination and hydrophilicity by calculating LogP for each of the crosslinked ODN/ORN products. Interestingly, the hydrophilicity of the ODN with inosine and ^OHV^K is highest among all the other ODNs, thus leading to the fastest deamination reaction (Figure 5A). The trend of hydrophilicity and the rate of deamination were found to be interrelated (Figure 5B). The main reason that we hypothesize here is that for a deamination reaction, it is important for the nucleophile, water, to attack the cross-linked cytosine for deamination to take place. Figure 6 depicts the proposed mechanism where the water attacks the c4 carbon of the cross-linked cytosine and for an effective attack, it is important that the core of the double helix should be able to provide a hydrophilic environment. Therefore, the most hydrophilic pair of counter bases and cross-linkers are able to achieve the highest rate of deamination.

Once it is established that hydrophilicity is one of the most important factors for the deamination of cross-linked cytosine in both DNA [31] and RNA, the major hurdle remaining was that the rate of deamination was still very low even with the most polar counter base and crosslinker pair with a half-life of ~4.5 days. For in vivo applications, this is not a feasible reaction time. Therefore, we then started to work on improving the reaction time of deamination.

**Figure 7:**
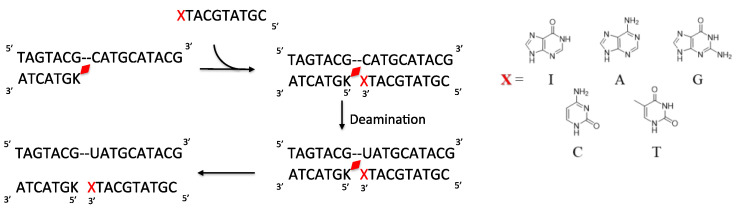
Scheme of deamination of the cytosine with disjointed complimentary ODNs. Here, K is ^CNV^K and X is the counter base of the target cytosine.

Thus, to drastically increase the hydrophilicity of the ODN containing the photo-crosslinker, the ODN was divided into two parts with the counter base of target cytosine in one ODN and the photo-crosslinker in the other ODN with a free phosphate at 3′-carbon of each of the counter bases (Figure 7 and Table 1). Then, following the deamination protocol, all three ODNs, namely, the target ODN(tC), the ODN(K) with ^CNV^K, and the ODN(X) with the counter bases A, T, G, C, or I or the ODN(Xp) with phosphate modified counter bases Ap, Tp, Gp, Cp, or Ip at a concentration of 5 µM each in 50 mM cacodylate buffer with 100 mM sodium chloride (pH 7.4) were irradiated with 385 nm radiation and then incubated for 24 h followed by photo-splitting with 312 nm radiation. The final product solution was analyzed with UPLC.

**Figure 8:**
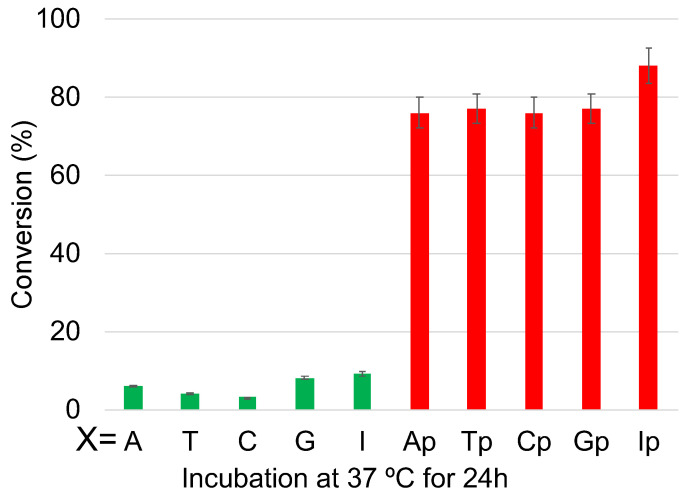
Conversion of cytosine to uracil after the photo-crosslink-assisted deamination reaction with ODN(K) and ODN(X/Xp).

The UPLC chromatograms showed some interesting results, wherein after 24 h, the ODNs without phosphate modification showed practically no deamination, whereas for those with phosphate modification, the reaction reached over 75% conversion within 24 h of incubation at 37 °C (Figure 8 and Figure S5). It should also be noted that the effect of counter-base inosine is still visible in both the phosphate-modified and non-modified ODNs compared to the other counter bases. This supports our hypothesis that the hydrophilicity of the counter base as well as the additional hydrophilicity provided by phosphate modification has a drastic effect on the rate of the deamination reaction.

It is important to note here that it was not possible to measure the LogP of the crosslinked ODNs to analyze the difference in the hydrophilicity of the counter bases as the ODN with counter bases is detached from the photo-crosslinker ODN; thus, it only provides the micro-environment in the hybridized state.

**Figure 9:**
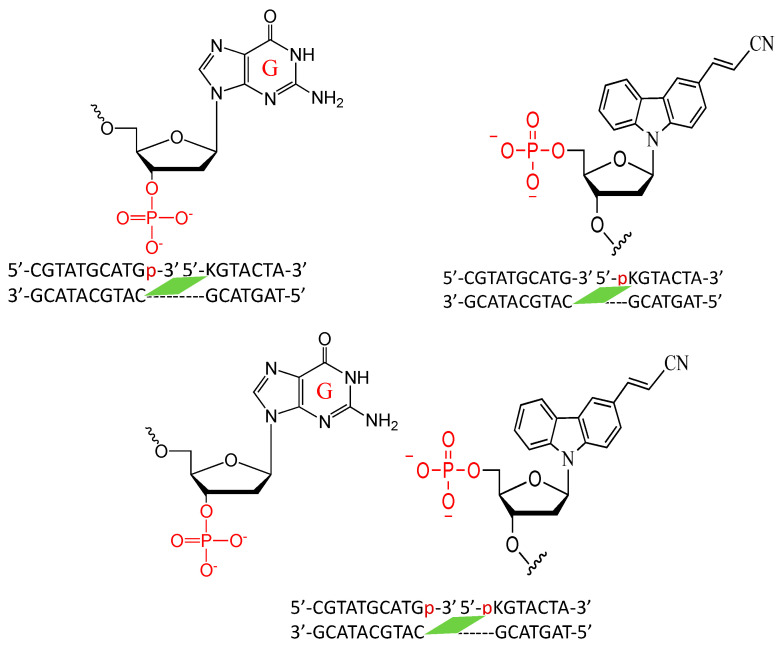
Sequence and structure of the disjointed ODNs with phosphate-modified guanine and ^CNV^K.

Next, we realized that even without using a hydrophilic counter base, it is possible to achieve high acceleration of the deamination reaction using the natural counter base of cytosine, namely guanine. Thus, to further accelerate the rate of the deamination reaction, we used only guanine as the counter base and ^CNV^K as the crosslinker, but in this instance, we studied the effect of phosphate modification on both the disjointed ODNs and compared it with single phosphate modification on either the counter base or the cross-linker ODN and no phosphate modification (Figure 9).

**Figure 10:**
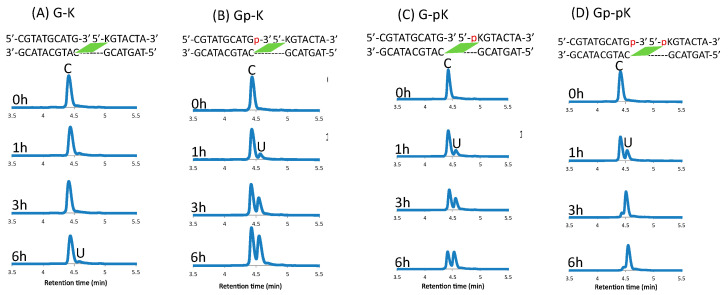
UPLC analysis of the deamination reaction of the photo-cross-linked duplex consisting of ODN(K/pK), ODN(X/Xp), and ODN(tC). [ODN(G/K<>C)] = 5 μM in 50 mM Na-cacodylate buffer (pH 7.4) containing 100 mM NaCl. incubated at 37 °C; photo splitting was performed with a transilluminator (312 nm) at 37 °C.

The crosslinked ODNs were incubated for 6 h at 37 °C in 50 mM cacodylate buffer with 100 mM sodium chloride (pH 7.4) and then photo-split using 312 nm radiation. The UPLC chromatograms are shown in Figure 10.

**Figure 11:**
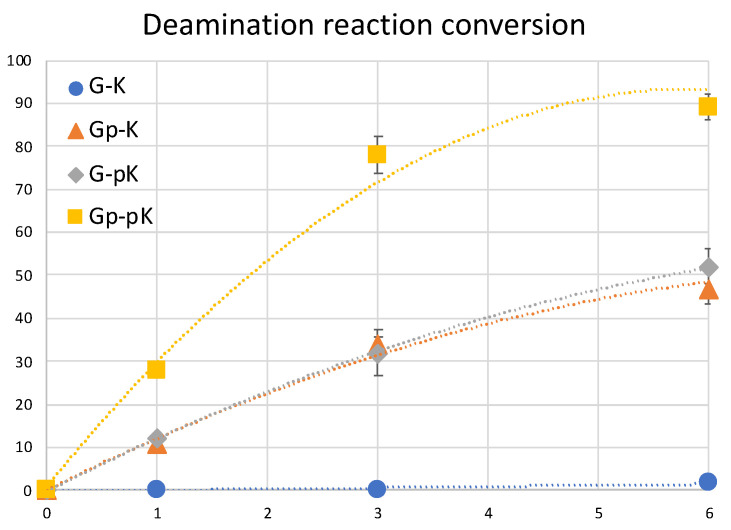
Conversion of cytosine to uracil after the photo-crosslink-assisted deamination reaction with ODN(K/pK) and ODN(X/Xp).

The difference between the phosphate-modified and non-modified ODNs are clearly visible, with almost 90% conversion of cytosine to uracil within 6 h of incubation at 37 °C when using the ODNs that are both modified with a phosphate group, whereas with single phosphate modification, the rate of conversion does not differ; although, without these modifications, the reaction does not proceed within this time frame (Figure 11).

**Figure 12:**
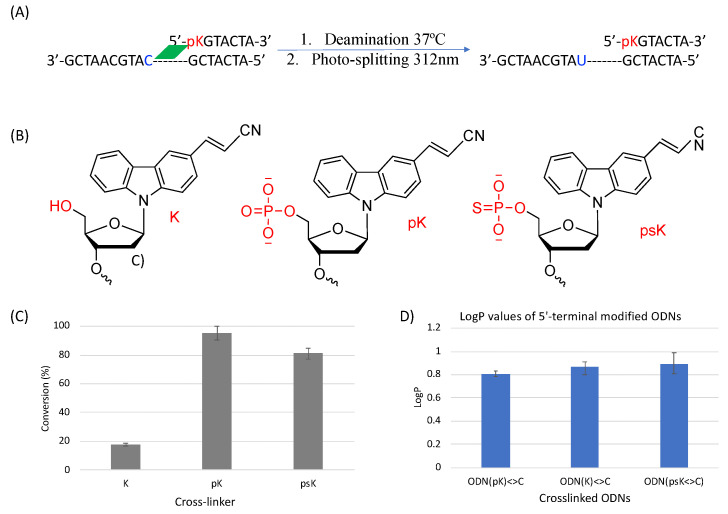
(**A**) Deamination reaction scheme with 5′-terminal modified ODNs. (**B**) Structure of the various 5′-terminal modifications. (**C**) Conversion ratio of cytosine to uracil using the different 5′-terminal modifications. (**D**) LogP values of the 5′-terminal modified ODNs.

To further clarify the effect of phosphate modification, we compared the deamination reaction of another 5′-terminal modified ODN which contains a sulfur atom replacing one of the oxygens of the phosphate group as shown in Figure 12. In this case, we used ^CNV^K crosslinkers without any phosphate modification, with phosphate modification, and with phosphorothioate modification to compare the deamination efficiency of cytosine when each of them are crosslinked to the target cytosine. It was interesting to observe that in both cases wherein a free phosphate group is present, the deamination reaction proceeded at ultra-fast speed, whereas with no free phosphate group, the reaction rate was rather slow. To understand the reason for the same outcomes, we tried to calculate the LogP values of these crosslinked ODNs as in our previous experiments with various crosslinkers and counterbases; hydrophilicity was a deciding factor in the deamination reaction rate.

**Figure 13:**
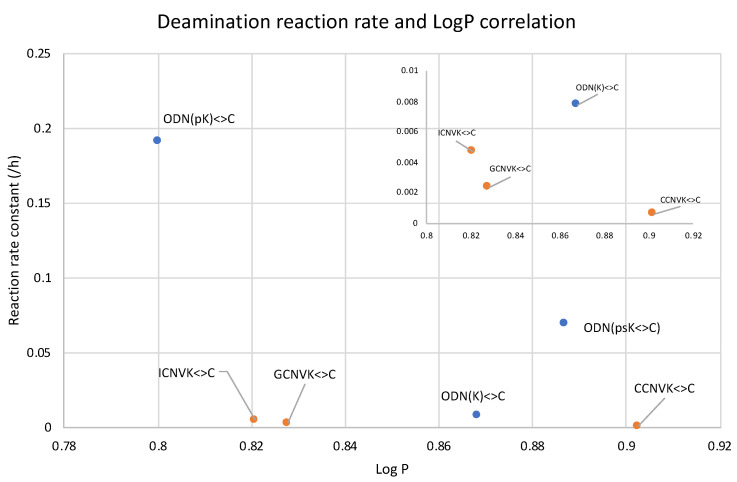
Compiled LogP values of the counterbase–crosslinker variation compared with 5′phosphate modification.

Upon observing the LogP values, the results obtained were vastly different from the expected results as the ODN(psK<>) had the highest LogP values, translating to the lowest hydrophilicity, but the reaction rate of ODN(psK<>C) was much higher than that of ODN(K<>C). On the other hand, ODN(pK<>C), with the highest hydrophilicity, had the highest reaction rate among all three ODNs. We then went on to compare our previous results with the current results of 5′-terminal modifications. Figure 13 shows the correlation of the deamination reaction rate with the LogP of the 5′-terminal modified ODNs and that of ORNs containing CNVK in the middle and different counter bases.

In Figure 13, it can be observed that the unmodified ODN(K<>C) with ^CNV^K at the 5′-terminal has a slightly higher reaction rate compared to the ODNs with ^CNV^K in the middle position despite being more hydrophobic than its counterpart with guanine as the counter base. On the other hand, for the ODNs with the free phosphate group at the 5′-terminal, the reaction rate is drastically higher than all other ODNs.

These results further confirm the fact that the deamination reaction acceleration is due to phosphate modifications and that it does not depend on higher hydrophilicity alone or only the freedom of movement provided by the disjoining of the ODNs in ODN(K<>C). Thus, we believe that hydrophilicity is not the only factor for accelerating the deamination reaction in the case of the 5′modified ODNs. To understand further, we tried to find the pKa values of these ODNs as the free phosphate group has an acidic proton which might have affected the reaction rate.

The pKa values of ODN(pK<>C) and ODN(psK<>C), which are depicted in Table 2, are an indication that at the physiological pH of 7.4, the phosphate group helps in the activation of incoming nucleophiles by possibly forming a hydrogen bond with the water molecule, thus providing better accessibility for the nucleophile to attack the target amino group of the cross-linked cytosine.

**Figure 14:**
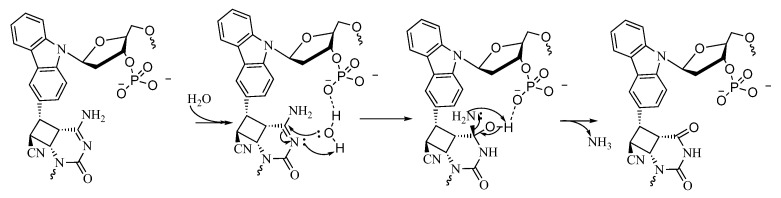
Proposed mechanism of the deamination of the cytosine cross-linked to the cross-linker in the complementary strand with a free phosphate group.

Based on these findings, we have proposed a mechanism wherein it is possible that the presence of a free phosphate group provides ease-of-access to the incoming nucleophile, water, by hydrogen bonding with the water molecule and thus facilitating the attack on the C4 carbon of the cytosine to afford deamination (Figure 14).

## 3. Materials and Methods

### 3.1. General

The 1H nuclear magnetic resonance (NMR) spectra were recorded on an AVANCE III 400 system (Bruker, Billerica, MA, USA). The mass spectra were recorded on a Voyager PRO-SF, (Applied Biosystems, Foster City, CA, USA). HPLC was performed on an InertSustain™ C18 column Cosmosil™ 5C_18_-AR-II column (5 μm, 10 × 150 mm, (Nacalai tesque, Kyoto, Japan) with JASCO PU-980, HG-980-31, and DG-980-50 systems equipped with a JASCO UV 970 detector (JASCO, Tokyo, Japan) at 260 nm. The reagents for DNA synthesis such as A, G, C, T-β-cyanoethyl phosphoramidite, and CPG support were purchased from Glen Research (Sterling, VA, USA).

### 3.2. ODN Synthesis

The phosphoramidite of ^CNV^K was prepared as per the protocol mentioned in the literature [38]. The ODNs containing ^TF^C were synthesized as per the method mentioned in the reference [37]. The ^IOHV^K, ^NH2V^K, and ^OMeV^K ODNs were synthesized by modification of the ODN containing ^OMeV^K by varying deprotection methods [31]. In the case of ^OHV^K, the oligonucleotide was deprotected using 0.4 M NaOH in an H_2_O and MeOH (1:4, *v*/*v*) ratio at 37 °C for 17 h. The oligonucleotides of ^NH2V^K and ^OMeV^K were deprotected using 28% ammonia solution at 65 °C for 4 h and 50 mM K_2_CO_3_ in MeOH at 37 °C for 12 h, respectively. The synthesized ODN were detached from the support through soaking in concentrated aqueous ammonia for 1 h at room temperature. Deprotection was conducted by heating the aqueous concentration for 4 h at 65 °C before removing the concentrated aqueous ammonia by speedvac and purifying the crude oligomer by reverse phase HPLC (with a flow rate of 3.0 mL/min, 60 °C) and lyophilizing it. Synthesis of the ODN was confirmed by MALDI-TOF-MS. The other ODNs were purchased from Fasmac (Kanagawa, Japan).

### 3.3. Isolation of the Photo-Cross-Linked dsDNA

The target ODN with the cytosine or its derivative (10 μM) and the ODN (XK), where X is one of the counter bases and K is the photo-cross-linker, (10 μM) in a buffer solution (100 mM NaCl, 50 mM sodium cacodylate, pH 7.4), were photo-irradiated at 385 nm for 180 s using UV-LED illuminator (Class 3B LED, MX302 OMRON Inc., Tokyo, Japan, 1600 mW cm^−2^) at 37 °C. In addition, the solution was purified by reverse phase HPLC, and the concentration of photo-cross-linked dsDNA was determined by absorbance at 260 nm.

### 3.4. Deamination

The 5 μM photo-cross-linked dsODN in the buffer solution (50 mM Na-cacodylate buffer (pH 7.4) containing 100 mM NaCl) was incubated at 90 °C for the cytosine derivatives or at 37 °C for all other cases.

### 3.5. Photo-Splitting and UPLC Analysis

After photo-splitting with irradiation of 312 nm (15 min at 37 °C, transilluminator with Longlife™ filter, (Funakoshi, Tokyo, Japan)), the reaction mixture was analyzed with a UPLC system (Aquity, Waters, Milord, MA, USA) fitted with an Acquity UPLC BEH C18 1.7 µm (2.1 × 50 mm) column; elution was with 0.05 M ammonium formate containing 3–6.5% CH_3_CN in 50 mM aqueous ammonium formate, linear-gradient (10 min), at a flow rate of 0.2 mL/min. The eluent was analyzed using UV absorbance at 260 nm.

### 3.6. Partition Coefficient (LogP)

The LogP of the photo-cross-linked DNA was measured by its retention time following the OECD protocol. Approximately 10 μM photo-cross-linked dsDNA was analyzed with an HPLC system; elution was with 0.05 M ammonium formate containing 98–50% CH_3_CN, linear-gradient (30 min), at a flow rate of 1 mL/min [39,40]. 4-acetylpyridine, aniline, acetanilide, phenol, benzonitrile, and acetophenone were used as the control compounds to create a calibration curve

### 3.7. Melting Point Analysis

The UV melting curve of the duplexes ODN(XK)/cODN(C) and the photo-cross-linked ODNs (5 µM in 50 mM Na-cacodylate buffer (pH 7.4) consisting of 100 mM NaCl) were measured (260 nm, 1 °C/min) by V-630bio spectrophotometer (Jasco, Japan), equipped with a temperature controller. The melting temperature (T_M_) of the duplex was calculated using the first derivative of the corresponding melting curve (Tables S7 and S8).

### 3.8. Molecular Modeling

Molecular modeling analysis was performed using Macromodel v8.1^20^ with AMBER* force field, water as the solvent, and the H-bond parameter 2.5 Å constrain. Energy minimization (500 iterations), both before and after stochastic molecular dynamics, was performed to find the most stable structure. The details of the molecular modeling simulations were described in the ESI.

## 4. Conclusions

We have demonstrated that hydrophilicity is an important factor for accelerating the deamination of cross-linked cytosine to uracil. The effect of hydrophilicity was shown at 90° using cytosine derivatives. Consecutively, the accelerating effect of hydrophilicity was also proven in RNA under physiological conditions. Furthermore, we were able to improve the deamination reaction rate from a half-life of a few days to a half-life of 1.5 h by using highly hydrophilic phosphate-modified ODNs. We further concluded that the effect which is responsible for the acceleration of the deamination of cytosine in the crosslinked ODNs is not only hydrophilicity but there are also other factors such as the protonation of free phosphate groups which help the incoming nucleophile to attack the amino group of the cytosine. Thus, in our future research, we plan to focus further on these effects.

Furthermore, phosphate modification was able to accelerate the deamination reaction drastically in vitro, but the applicability of this strategy to be used in vivo is still unknown.

## Figures and Tables

**Table 1:** Sequence of the ODNs with and without phosphate modification: ODN(K) and the target ODN (tC).

ODN(X) X=	Sequence (5′→3′)	ODN(X) X=	Sequence (5→3′)
G	CGTATGCATG	Gp	CGTATGCATGp
C	CGTATGCATC	Cp	CGTATGCATCp
T	CGTATGCATT	Tp	CGTATGCATTp
A	CGTATGCATA	Ap	CGTATGCATAp
I	CGTATGCATI	Ip	CGTATGCATIp
ODN(K)	^CNV^KGTACTA	ODN (tC)	TAGTACGCATGCATACG

p: free phosphate group.

**Table 2:** The acid dissociation constant (pKa) and deamination rate constant of phosphate and phosphorothioate modified ODNs.

ODN	pKa	Deamination Reaction Rate Constant (h^−1^)
ODN(pK<>C)	6.5	0.1919
ODN(psK<>C)	5.0	0.0692
ODN(K<>C)	-	0.0078

## Data Availability

Data is contained within the article or Supplementary Material.

## Supplementary Materials

The following supporting information can be downloaded at: https://www.mdpi.com/article/10.3390/cimb45060298/s1. Table S1: MALDI-TOF-MS analysis of synthesized ODNs with modified cytosine; Table S2: MALDI-TOF-MS analysis of synthesized ODNs; Table S3: MALDI-TOF-MS analysis of cross-linked ODN/ORN; Table S4: MALDI-TOF-MS analysis of cross-linked ODN/ORN; Figure S1. LogP analysis of photo-cross-linked duplex containing cytosine derivatives by water-octanol method. * Shows peak of photo-cross-linked duplex. The plots are result of HPLC analysis; Figure S2. UPLC analysis of deamination product after photo-splitting of ODN with inosine as counter base; * indicates new peaks corresponding to cORN(U).; Figure S3. UPLC analysis of deamination product after photo-splitting of ODN with guanine as counter base; * indicates new peaks corresponding to cORN(U).; Figure S4. UPLC analysis of deamination product after photo-splitting of ODN with cytosine as counter base; * indicates new peaks corresponding to cORN(U).; Table S5: Log P values corresponding to figure 5A of main text; Figure S5. UPLC analysis of the deamination reaction of the photo-cross-linked duplex consisting of ODN(K), A) ODN(X); B) ODN(Xp) and cODN(C). [ODN(XK<>C)] = 5 μM in 50 mM Na-cacodylate buffer (pH 7.4) containing 100 mM NaCl. incubated at 37 °C, photo splitting was performed with transilluminator (312 nm) at 37 °C.; Table S6: Melting temperature of duplex with modified cytosine; Table S7: Melting temperature of ODN/ORN dplex with CNVK; Table S8: Melting temperature of ODNs with phosphate modification; Molecular modeling details.

## References

[1] Ikebe S., Tanaka M., Ozawa T. (1995). Point mutations of mitochondrial genome in Parkinson’s disease. Mol. Brain Res..

[2] Sakuraba H., Oshima A., Fukuhara Y., Shimmoto, Nagao Y., Bishop D.F., Desnick R.J., Suzuki Y. (1990). Identification of point mutations in the alpha-galactosidase A gene in classical and atypical hemizygotes with Fabry disease. Am. J. Hum. Genet..

[3] Li J., Uversky V.N., Fink A.L. (2001). Effect of familial Parkinson’s disease point mutations A30P and A53T on the structural properties, aggregation, and fibrillation of human alpha-synuclein. Biochemistry.

[4] De Vries D.D., van Engelen B.G., Gabreëls F.J., Ruitenbeek W., van Oost B.A. (1993). A second missense mutation in the mitochondrial ATPase 6 gene in Leigh’s syndrome. Ann. Neurol..

[5] Lake N.J., Compton A.G., Rahman, Thorburn D.R. (2016). Leigh syndrome: One disorder, more than 75 monogenic causes. Ann. Neurol..

[6] Baertling F., Rodenburg R.J., Schaper J., Smeitink J., Koopman W.J.H., Mayatepek E., Morava E., Distelmaier F. (2014). A guide to diagnosis and treatment of Leigh syndrome. J. Neurol. Neurosurg. Psychiatry.

[7] Cascalho M. (2004). Advantages and disadvantages of cytidine deamination. J. Immunol..

[8] Lamont P.J., Surtees R., Woodward C.E., Leonard J.V., Wood N.V., Harding A.E. (1998). Clinical and laboratory findings in referrals for mitochondrial DNA analysis. Arch. Dis. Child..

[9] Watanabe M., Nobuta A., Tanaka J., Asaka M. (1996). An effect of K-ras gene mutation on epidermal growth factor receptor signal transduction in PANC-1 pancreatic carcinoma cells. Int. J. Cancer.

[10] Storici F., Lewis L.K., Resnick M.A. (2001). In vivo site-directed mutagenesis using oligonucleotides. Nat. Biotechnol..

[11] Puchta H., Fauser F. (2013). Gene targeting in plants: 25 years later. Int. J. Dev. Biol..

[12] Tan W.S., Carlson D.F., Walton M.W., Fahrenkrug S.C., Hackett P.B. (2012). Precision editing of large animal genomes. Adv. Genet..

[13] Jiang W., Bikard D., Cox D., Zhang F., Marraffini L.A. (2013). RNA-guided editing of bacterial genomes using CRISPR-Cas systems. Nat. Biotechnol..

[14] Papathanasiou S., Markoulaki S., Blaine L., Leibowitz M., Zhang C.Z., Jaenisch R., Pellman D. (2021). Whole chromosome loss and genomic instability in mouse embryos after CRISPR-Cas9 genome editing. Nat. Commun..

[15] Doudna J., Charpentier E. (2014). The new frontier of genome engineering with CRISPR-Cas9. Science.

[16] White M.K., Kaminski R., Young W.B., Roehm P.C., Khalili K. (2017). CRISPR Editing Technology in Biological and Biomedical Investigation. J. Cell. Biochem..

[17] Grünewald J., Zhou R., Garcia S., Iyer S., Lareau C., Aryee M., Joung J. (2019). Transcriptome-wide off-target RNA editing induced by CRISPR-guided DNA base editors. Nature.

[18] Bhakta S., Tsukahara T. (2021). Double MS2 guided restoration of genetic code in amber (TAG), opal (TGA) and ochre (TAA) stop codon. Enzyme Microb. Technol..

[19] Fan J., Ding Y., Ren C., Song Z., Yuan J., Chen Q., Du C., Li C., Wang X., Shu W. (2021). Cytosine and adenine deaminase base-editors induce broad and nonspecific changes in gene expression and splicing. Commun. Biol..

[20] Frommer M., McDonald L.E., Millar D.S., Collis C.M., Watt F., Grigg G.W., Molloy P.L., Paul C.L. (1992). A genomic sequencing protocol that yields a positive display of 5-methylcytosine residues in individual DNA strands. Proc. Natl. Acad. Sci. USA.

[21] Yamana K., Yoshikawa A., Nakano H. (1996). Synthesis of a new photoisomerizable linker for connecting two oligonucleotide segments. Tetrahedron Lett..

[22] Lee B.L., Blake K.R., Miller P.S. (1988). Interaction of psoralen-derivatized oligodeoxyribonucleoside methylphosphonates with synthetic DNA containing a promoter for T7 RNA polymerase. Nucleic Acids Res..

[23] Kurz M., Gu K., Lohse P.A. (2000). Psoralen photo-crosslinked mRNA-puromycin conjugates: A novel template for the rapid and facile preparation of mRNA-protein fusions. Nucleic Acids Res..

[24] Liang X., Wakuda R., Fujioka K., Asanuma H. (2010). Photoregulation of DNA transcription by using photoresponsive T7 promoters and clarification of its mechanism. FEBS J..

[25] Iwase R., Namba M., Yamaoka T., Murakami A. (1997). Gene regulation by decoy approach (I): Synthesis and properties of photo-crosslinked oligonucleotides. Nucleic Acids Symp. Ser..

[26] Fujimoto K., Konishi-Hiratsuka K., Sakamoto T., Yoshimura Y. (2010). Site-specific photochemical RNA editing. Chem. Commun..

[27] Fujimoto K., Konishi-Hiratsuka K., Sakamoto T., Yoshimura Y. (2010). Site-Specific Cytosine to Uracil Transition by Using Reversible DNA Photo-crosslinking. ChemBioChem.

[28] Kreutzer D.A., Essigmann J.M. (1998). Oxidized, deaminated cytosines are a source of C –> T transitions in vivo. Proc. Natl. Acad. Sci. USA.

[29] Sethi S., Ooe M., Sakamoto T., Fujimoto K. (2017). Effect of nucleobase change on cytosine deamination through DNA photo-cross-linking reaction via 3-cyanovinylcarbazole nucleoside. Mol. BioSyst..

[30] Sethi S., Yasuharu T., Nakamura S., Fujimoto K. (2017). Effect of substitution of photo-cross-linker in photochemical cytosine to uracil transition in DNA. Bioorg. Med. Chem. Lett..

[31] Sethi S., Nakamura S., Fujimoto K. (2018). Study of Photochemical Cytosine to Uracil Transition via Ultrafast Photo-Cross-Linking Using Vinylcarbazole Derivatives in Duplex DNA. Molecules.

[32] Peng W., Shaw B.R. (1886). Accelerated deamination of cytosine residues in UV-induced cyclobutane pyrimidine dimers leads to CC→TT transitions. Biochemistry.

[33] Tu Y., Dammann R., Pfeifer G.P. (1998). Sequence and time-dependent deamination of cytosine bases in UVB-induced cyclobutane pyrimidine dimers in vivo. J. Mol. Biol..

[34] Cannistraro V.J., Pondugula S., Song Q., Taylor J.S. (2015). Rapid deamination of cyclobutane pyrimidine dimer photoproducts at TCG sites in a translationally and rotationally positioned nucleosome in vivo. J. Biol. Chem..

[35] Bird A.P. (1986). CpG-rich islands and the function of DNA methylation. Nature.

[36] Skirmantas K., Nathaniel H. (2009). The nuclear DNA base 5-hydroxymethylcytosine is present in Purkinje neurons and the brain. Science.

[37] Nakamura S., Yang H., Hirata C., Kersaudy F., Fujimoto K. (2017). Development of 19F-NMR chemical shift detection of DNA B–Z equilibrium using 19F-NMR. Org. Biomol. Chem..

[38] Yoshimura Y., Fujimoto K. (2008). Ultrafast Reversible Photocrosslinking Reaction: Toward in Situ DNA Manipulation. Org. Lett..

[39] OECD (2022). Test No. 117: Partition Coefficient (n-Octanol/Water), HPLC Method, OECD Guidelines for the Testing of Chemicals, Section 1.

[40] Henczi M., Nagy J., Weaver D.F. (1995). Determination of octanol-water partition coefficients by an HPLC method for anticonvulsant structure-activity studies. J. Pharm. Pharmacol..

