# Supplementary material for: Acceleration of the Deamination of Cytosine through Photo-Crosslinking
Source: Curr Issues Mol Biol. 2023 May 29;45(6):4687–700. doi: 10.3390/cimb45060298 (PMC10297672; doi:10.3390/cimb45060298)
Supplement: Supplementary file 1 [file cimb-45-00298-s001.zip › cimb-2359134-supplementary.pdf]

Supplementary information

Acceleration of deamination of cytosine through photo-crosslinking

Siddhant Sethi [1], Yasuharu Takashima[1], Shigetaka Nakamura[1], Licheng Wan[1], Nozomi Honda[1], Kenzo Fujimoto [1,*]

1. Bioscience, Biotechnology, and Biomedical Engineering Research Area
Japan Advanced Institute of Science and Technology
Asahi-dai 1-1, Nomi, Ishikawa, 923-1292, Japan; siddhant@jaist.ac.jp
****Correspondence: kenzo@jaist.ac.jp; Tel.: +81-761-51-1673***

1. MALDI-TOF-MS analysis

Table S1: MALDI-TOF-MS analysis of synthesized ODNs with modified cytosine.

Entry	Sequence(5'-3')	Found	Calcd [M+H]+
ODN(K)	TGCG^{CNV}KCCGT	2796.99	2797.52
cODN(C)	ACGGACGCA	2733.02	2732.52
cODN(^{m}C)	ACGGA^{m}CGCA	2746.80	2746.53
cODN(^{hm}C)	ACGGA^{hm}CGCA	2753.42	2752.53
cODN(^{tf}C)	ACGGA^{tf}CGCA	2758.83	2758.53

Table S2: MALDI-TOF-MS analysis of synthesized ODNs

Entry	Sequence	Found	Calcd [M+H]+
ODN(GCNVK)	AAATGCGCNVKACGTCCC	4628.11	4627.84
ODN(GOHVK)	AAATGCGOHVKACGTCCC	4632.98	4632.08
ODN(GOMeVK)	AAATGCGOMeVKACGTCCC	4661.39	4661.3
ODN(GNH2VK)	AAATGCGNH2VKACGTCCC	4645.02	4645.33
ODN(ICNVK)	AAATGCICNVKACGTCCC	4611.53	4610.97
ODN(IOHVK)	AAATGCIOHVKACGTCCC	4617.04	4617.17
ODN(IOMeVK)	AAATGCIOMeVKACGTCCC	4646.05	4645.62
ODN(INH2VK)	AAATGCINH2VKACGTCCC	4630	4629.65
ODN(CCNVK)	AAATGCCCNVKACGTCCC	4588.23	4587.84
ODN(COHVK)	AAATGCCOHVKACGTCCC	4592.99	4592.87
ODN(COMeVK)	AAATGCCOMeVKACGTCCC	4621.74	4621.19
ODN(CNH2VK)	AAATGCCNH2VKACGTCCC	4605.28	4605.22

Table S3: MALDI-TOF-MS analysis of cross-linked ODN/ORN

**Entry**	**Found**	**Calcd [M+H]+**
ODN(GCNVK)/cORN(C)	9435.49	9433.49
ODN(GOHVK)/cORN(C)	9438.38	9437.73
ODN(GOMeVK)/cORN(C)	9468.02	9466.95
ODN(GNH2VK)/cORN(C)	9452.3	9450.98
ODN(ICNVK)/cORN(C)	9417.99	9416.62
ODN(IOHVK)/cORN(C)	9424.07	9422.82
ODN(IOMeVK)/cORN(C)	9452.58	9451.27
ODN(INH2VK)/cORN(C)	9438.02	9435.3
ODN(CCNVK)/cORN(C)	9395.92	9393.49
ODN(COHVK)/cORN(C)	9400.31	9398.52
ODN(COMeVK)/cORN(C)	9427.33	9426.84
ODN(CNH2VK)/cORN(C)	9429.44	9410.87

Table S4: MALDI-TOF-MS analysis of cross-linked ODN/ORN

**Entry**	**Found**	**Calcd [M+H]+**
ODN(GCNVK)/cORN(C)	9435.49	9433.49
ODN(GOHVK)/cORN(C)	9438.38	9437.73
ODN(GOMeVK)/cORN(C)	9468.02	9466.95
ODN(GNH2VK)/cORN(C)	9452.3	9450.98
ODN(ICNVK)/cORN(C)	9417.99	9416.62
ODN(IOHVK)/cORN(C)	9424.07	9422.82
ODN(IOMeVK)/cORN(C)	9452.58	9451.27
ODN(INH2VK)/cORN(C)	9438.02	9435.3
ODN(CCNVK)/cORN(C)	9395.92	9393.49
ODN(COHVK)/cORN(C)	9400.31	9398.52
ODN(COMeVK)/cORN(C)	9427.33	9426.84
ODN(CNH2VK)/cORN(C)	9429.44	9410.87

2. Log P analysis

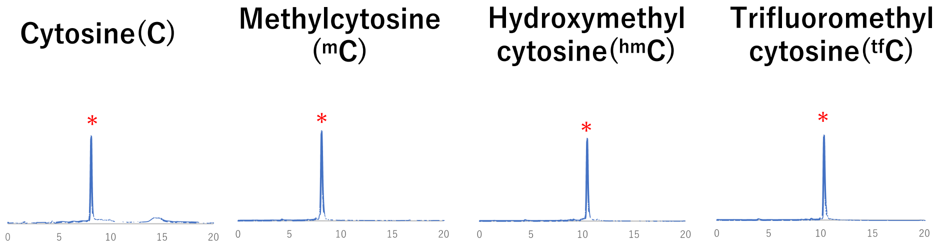

Figure S1. LogP analysis of photo-cross-linked duplex containing cytosine derivatives by water-octanol method. * Shows peak of photo-cross-linked duplex. The plots are result of HPLC analysis.

3. UPLC analysis of deamination by using varying photo-crosslinkers and counter bases

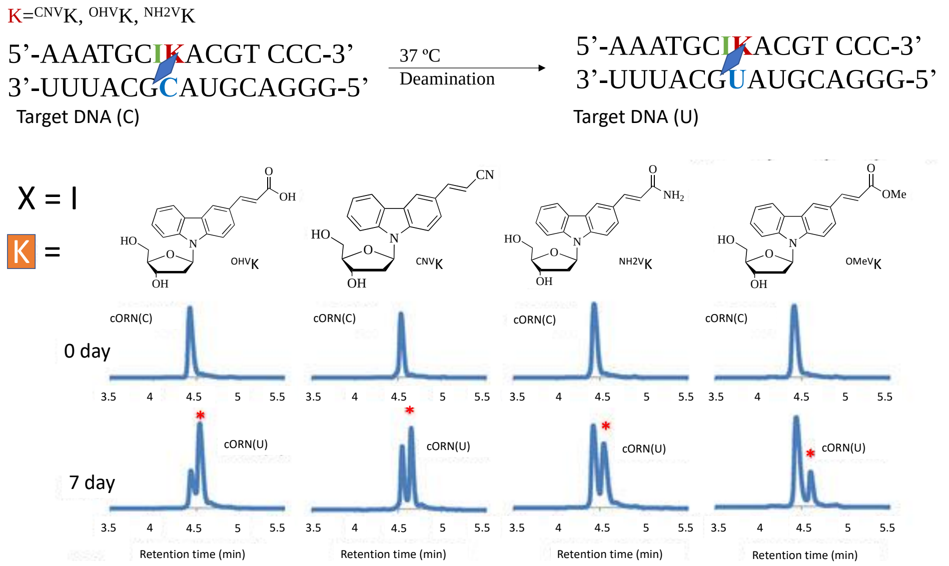

Figure S2. UPLC analysis of deamination product after photo-splitting of ODN with inosine as counter base; * indicates new peaks corresponding to cORN(U).

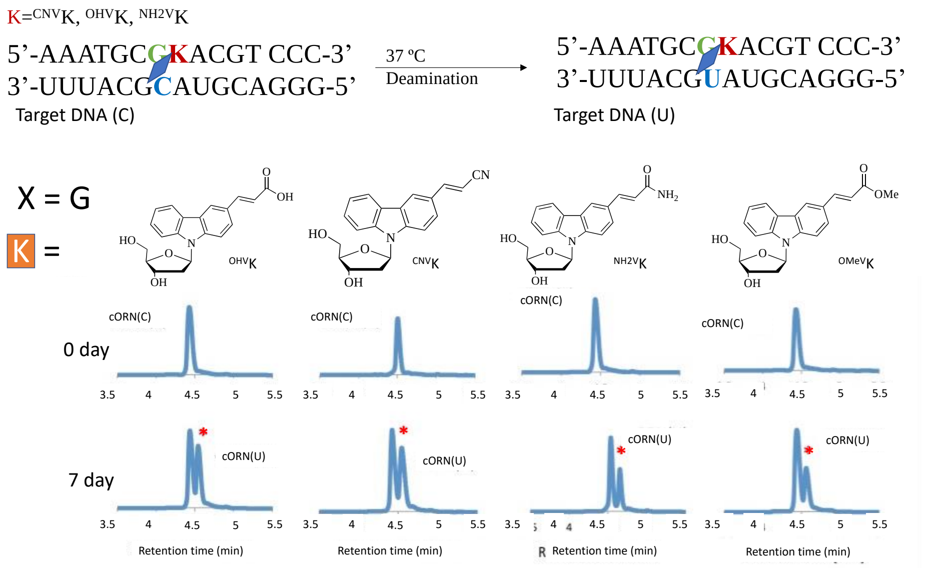

Figure S3. UPLC analysis of deamination product after photo-splitting of ODN with guanine as counter base; * indicates new peaks corresponding to cORN(U).

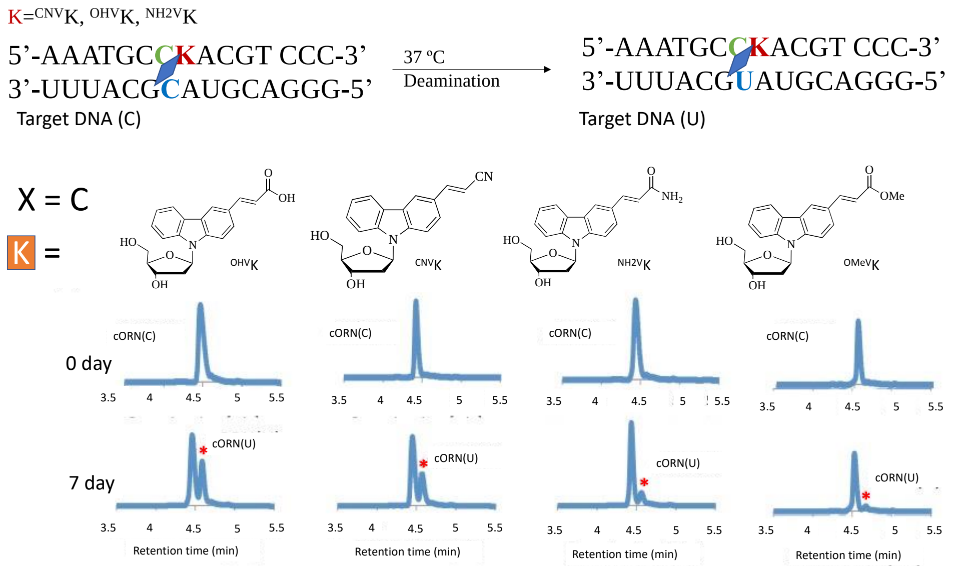

Figure S4. UPLC analysis of deamination product after photo-splitting of ODN with cytosine as counter base; * indicates new peaks corresponding to cORN(U).

4. Log P values of crosslinked ODN with varying crosslinker and counter base

Table S5: Log P values corresponding to figure 5A of main text

		Crosslinker			
		OHVK	NH2VK	CNVK	OMeVK
Counter base	Inosine	0.558513	0.713106	0.8208	0.76
	Guanine	0.640152	0.761742	0.827748	0.77
	Cytosine	0.700947	1.129986	0.902439	0.79

5. UPLC chromatograms with phosphate group modificated ODN for deamination analysis

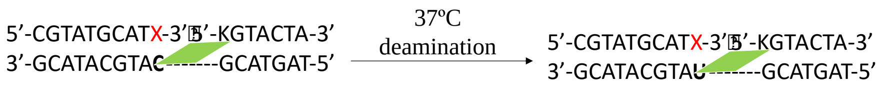

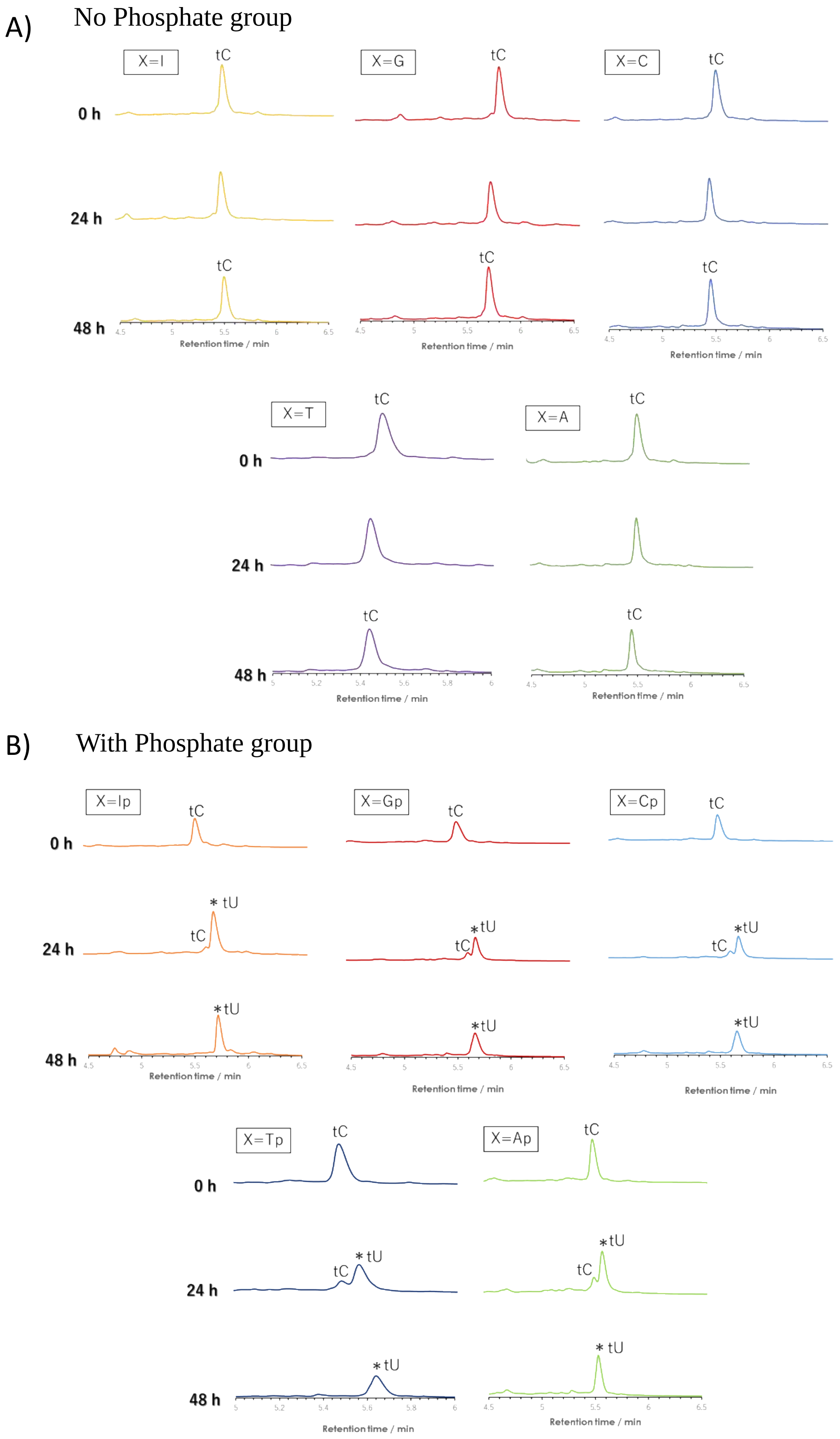

Figure S5. UPLC analysis of the deamination reaction of the photo-cross-linked duplex consisting of ODN(K), A) ODN(X); B) ODN(Xp) and cODN(C). [ODN(XK<>C)] = 5 μM in 50 mM Na-cacodylate buffer (pH 7.4) containing 100 mM NaCl. incubated at 37 °C, photo splitting was performed with transilluminator (312 nm) at 37 °C.

6. Melting temperature analysis

Table S4: Melting temperature of duplex with modified cytosine

Entry	Melting temperature
ODN(CNVK)/ODN(C)	29.8 °C
ODN(CNVK)/ODN(mC)	33.8 °C
ODN(CNVK)/ODN(hmC)	31.6 °C
ODN(CNVK)/ODN(tfC)	28.8 °C

Table S5: Melting temperature of ODN/ORN dplex with CNVK

Entry	Tm
ODN(GCNVK)/cORN(C)	55.4°C
ODN(ICNVK)/cORN(C)	50.5°C
ODN(CCNVK)/cORN(C)	46.1°C

Table S6: Melting temperature of ODNs with phosphate modification

Entry	Tm (ºC)
ODN(K)+ODN(C)	32.3
ODN(pK)+ODN(C)	35.4
ODN(K<>C)	49.9
ODN(pK<>C)	43.6

7. Molecular modeling

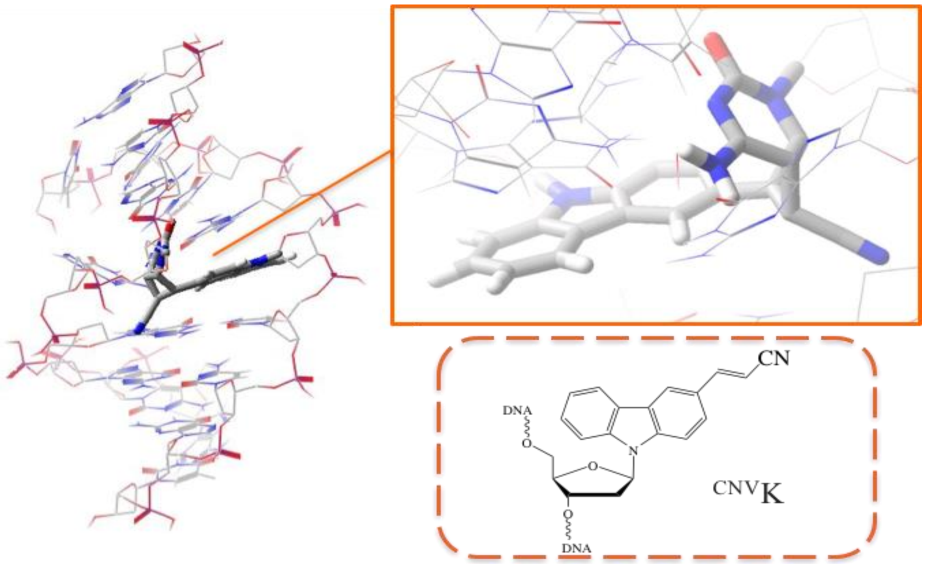

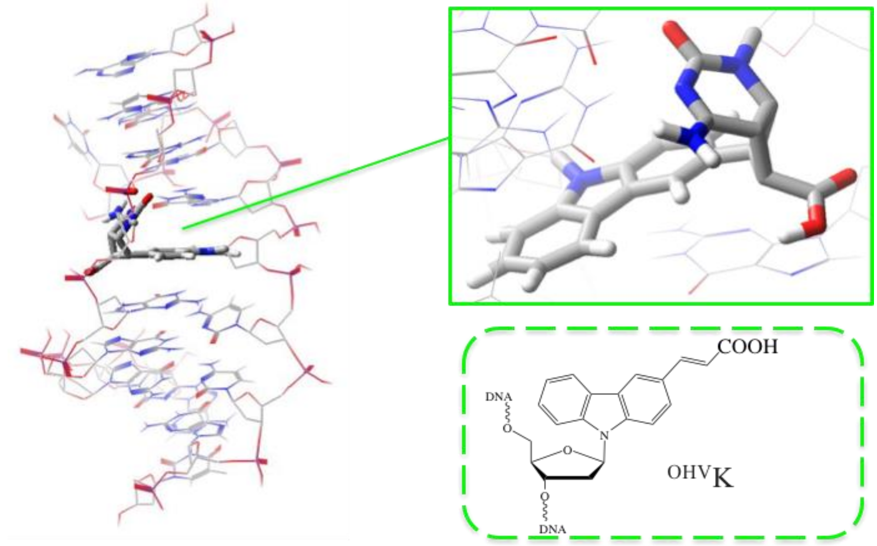

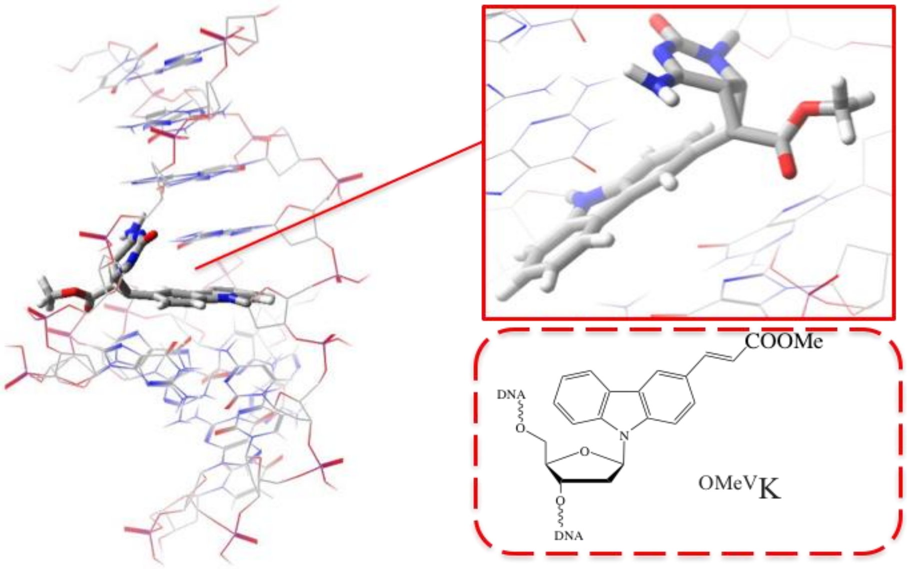
COOMe
DNA
O
N
O
O
DNA
OMeVK

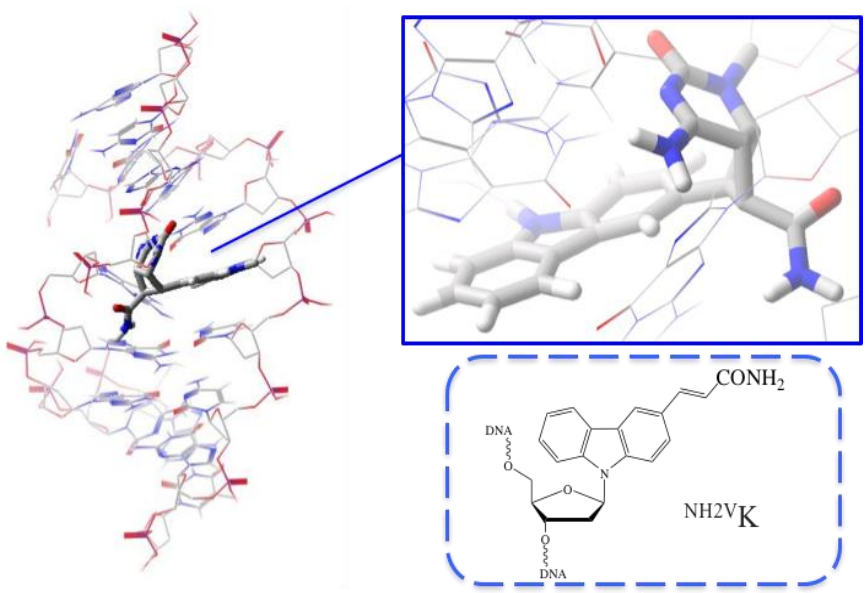
CONH2
DNA
O
N
O
O
DNA
NH2VK

Molecular modeling experiment details

Macromodel v8.1 Maestro v5.106 ©Schrodinger LLC
Dynamics method: Stochastic, no shaking
Simulation temperature: 300 K
Time step: 1.500 fs
Equilibriation time: 1.0 ps
Simulation time: 10.0 ps
Minimization method: PRCG
Max # of iterations: 500
Converge on: Gradient
Convergence threshold: 0.0500
Force field: AMBER*
Solvent: Water
Electrostatic treatment: constant dielectric
Dielectric constant: 1.0
Charges from: force field;
extended cut off Vanderwaals: 8.0 Electrostatic: 20.0 H-bond: 4.0
